# Supplementary material for: Combination of Expanded Allogeneic NK Cells and T Cell-Based Immunotherapy Exert Enhanced Antitumor Effects
Source: Cancers (Basel). 2022 Dec 30;15(1):251. doi: 10.3390/cancers15010251 (PMC9818244; doi:10.3390/cancers15010251)
Supplement: Supplementary file 1 [file cancers-15-00251-s001.zip › Supplementary Tables.zip!/Supplementary Table S1.pdf]

Supplementary Table S1. The HLA allele information.

	Donor.No	HLA-A	HLA-B	HLA-C	HLA-DR	HLA-DQ
PBMC/T	P1	02:01,11:01	40:01,67:01	07:02,07:02	12:02,16:02	05:02,03:01
	P2	01:01,30:01	13:02,35:01	04:01,06:02	01:01,07:01	05:01,02:02
	P3	30:01,32:01	13:02,44:02	05:01,06:02	07:01,12:01	02:02,03:01
	P4	02:01,11:01	40:01,54:01	01:02,03:04	04:03,08:03	06:01,03:02
	P5	02:10,31:01	15:58,40:06	01:02,08:01	12:01,14:54	05:02,03:01
	P6	11:01,31:01	51:01,51:02	14:02,15:02	12:10,15:01	03:01,06:01
NK	N1	02:01,24:02	15:27,35:01	03:03,04:01	08:03,15:01	06:01,06:02
	N2	11:01,11:01	15:01,40:01	04:01,07:02	04:03,04:06	03:02,03:02
	N3	11:01,26:01	40:01,40:01	07:02,07:66	14:54,14:54	05:02,05:02
	N4	24:02,24:02	46:01,51:02	01:02,15:02	08:02,09:01	03:03,04:02
	N5	01:01;30:01	13:02;37:01	06:02;06:02	07:01;10:01	05:01;02:02
